# Supplementary material for: Integration of Multiple Resolution Data in 3D Chromatin Reconstruction Using ChromStruct
Source: Biology (Basel). 2021 Apr 16;10(4):338. doi: 10.3390/biology10040338 (PMC8072831; doi:10.3390/biology10040338)

CHROMSTRUCT v4.3 folder: README

This folder contains 8 files:

- `ChromStruct_4.3.py` (Command-line version of CHROMSTRUCT 4.3)
- `Plot_Energy_Chain_2.0.py` (Command-line code to display CHROMSTRUCT results)
- `ChromStruct_4.3_GUI.py` (User-interface version of CHROMSTRUCT 4.3)
- `README.pdf` (this file)
- Four input files to be used as example: `HiC_2Mb_5kb.txt` (HiC contact matrix of human hematopoietic cells, chromosome 12 [5Mb-7Mb] at 5kb resolution, from [5]), H3K27me3_2Mb.txt (ChIPseq data referred to the presence H3K27me3 methylation, related to `HiC_2Mb_5kb.txt`, from [5]), ActiveGenes_2Mb.txt (RNAseq data referred to the presence of active genes, related to `HiC_2Mb_5kb.txt`, from [5]), ctcf_2Mb.txt (CTCF ChIA-PET data related to `HiC_2Mb_5kb.txt`, from [5,6]).

The code files are all self-contained and only need a Python 3 interpreter to run.

Dependencies:

- `Python 3`
- `Numpy`
- `Scipy`
- `Matplotlib`
- `Pyquaternion`
- `Tkinter (for the GUI)`

ChromStruct_4.3.py

The code can be run from the interactive python dialog or from the console window, by invoking:

```
> python ChromStruct_4.3.py
```

The program will ask on the interactive python dialog to insert the names of following files:

- `> File name (.txt) = ?` **Hi-C square contact frequency matrix** in txt format (Example: `HiC_2Mb_5kb.txt`).
- `> RNAseq file(.txt) = ?` (Optional) **RNA-seq data** in txt format: binary array at the same resolution and same dimension of the contact matrix. “1” if the bin is interested by expressed genes, “0” if the bin is not.
- `> ChIPseq file(.txt) = ?` (Optional) **ChIP-seq data associated to H3K27me3 methylation** (or other histone modification associated to repression and compaction) in txt format: binary matrix at the same resolution and same dimension of the contact matrix. “1” if the bin is interested by H3K27me3 methylation (cut off 300), “0” if the bin is not. The matrix, inside the

program, is multiplied by **100** and added to the HiC contact matrix, in order to strengthen the proximity constraint related to the binding sites.

- >CTCF file(.txt) = ? (Optional) **CTCF ChIA-PET data** in txt format: binary square matrix at the same resolution and same dimension of the contact matrix. “1” if the couple of bins is interested by a contact, “0” if not.

<u>*IMPORTANT:* Remember to set the variable RIS as the resolution in kb of the input contact matrix. (In the example RIS=5).</u>

If the data file is:

filename.suffix

the code produces a number of files with these names:

- filename_<timestamp>_<level>_BlockSizes.txt: a list with as many entries as blocks detected at resolution level <level>. <level> is coded as an integer from 0 to *number of detected levels – 1*. <timestamp>, a character string formatted as <yy-mm-dd-hhmm>, is referred to the date and time when the algorithm starts, and is used to identify the files coming from the same data file and the same run.

- filename_<timestamp>_Log.txt: a self-explanatory log file.

- filename_<timestamp>_<level>_<block>_Energy.txt: a real array with 3 columns and as many rows as accepted annealing updates of the configuration of block <block> (coded as an integer from 0 to *number of detected blocks at level* <level> *– 1*) at the resolution level <level>. The first real in each row is the data fit part of the score function, the second is the constraint part, and the third is the total score. If the related checkbox in the GUI is active, this is plotted as soon as the iteration at each block and level is complete. It can also be plotted by Plot_Energy_Chain_2.0.py.

- filename_<timestamp>_<level>_<block>.txt: a real array with 4 columns and as many rows as three times the number of beads in block <block> at level <level>. The first of each three rows contains the coordinates x, y and z (in nm) of the first endpoint of a bead; the second contains the coordinates of the centroid, and the third contains the coordinates of the second endpoint. Each row is completed with the estimated size (in nm) of the related bead. If the related checkbox in the GUI is active, this structure is plotted as soon as block <block> at level <level> has been computed. It can also be plotted by Plot_Energy_Chain_2.0.py.

- filename_<timestamp>_LastConf.txt: a real array with the same format as filename_<timestamp>_<level>_<block>.txt, with the final 3D chain configuration. This is plotted at the end of the procedure. It can also be plotted by Plot_Energy_Chain_2.0.py.

- filename_<timestamp>_DistMat.txt: a real array with the mutual distances between bead centroids, computed from the final estimated structure in filename_<timestamp>_LastConf.txt.

The code also prints the logfile information in the console window (score values and related annealing temperature once in every 1000 cycles). To close the program after the final plot, close the plot window and then the graphical interface. To abort the program, press <ctrl> + c from the keyboard.

Plot_Energy_Chain_2.0.py

The code can be run from the interactive python dialog or from the console window, by invoking:

`>python Plot_Energy_Chain_2.0.py`

The program will ask on the interactive python dialog to insert the names of following files:

- `>Chain: file name = ?` **filename_<timestamp>_LastConf.txt** produced by `ChromStruct_4.3.py`: the final configuration is represented by a red smooth curve linking the centroids of the bins (smoothing is obtained by cubic spline interpolation).

- `> Chain: centromeres file name = ?` (Optional) **filename_<timestamp>_centromere.txt** produced by `ChromStruct_4.3.py`: the centromere region is emphasised in green.

- `>Chain: telomeres file name = ?` (Optional) **filename_<timestamp>_telomeres.txt** produced by `ChromStruct_4.3.py`: the telomeric regions are emphasised in blue.

- Sphere envelops are set with default "not" (`ball='n'`). To display each bin as a sphere set `ball='y'` at line 108.

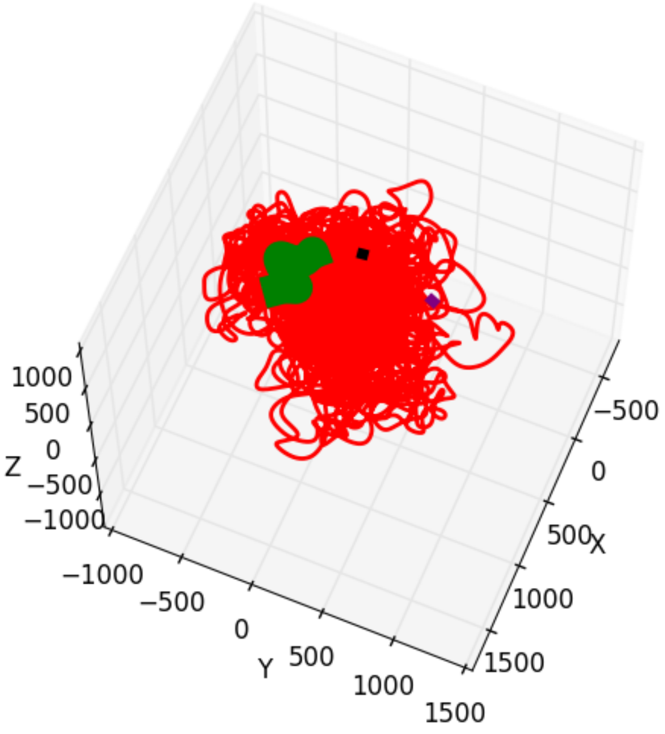

Output of Chromosome 16 at 25 kb resolution (GEO GM06990) produced by `Plot_Energy_Chain_2.0.py` : smooth red line represents final configuration produced by `ChromStruct_4.3.py`, starting point in black, end point in purple, centromere in green.

ChromStruct_4.3_GUI.py

The code can be run from the interactive python dialog or from the console window, by invoking:

```
> python ChromStruct_4.3_GUI.py
```

The ChromStruct GUI (version 4.3) appears as shown below. All the parameters can be edited before starting the program.

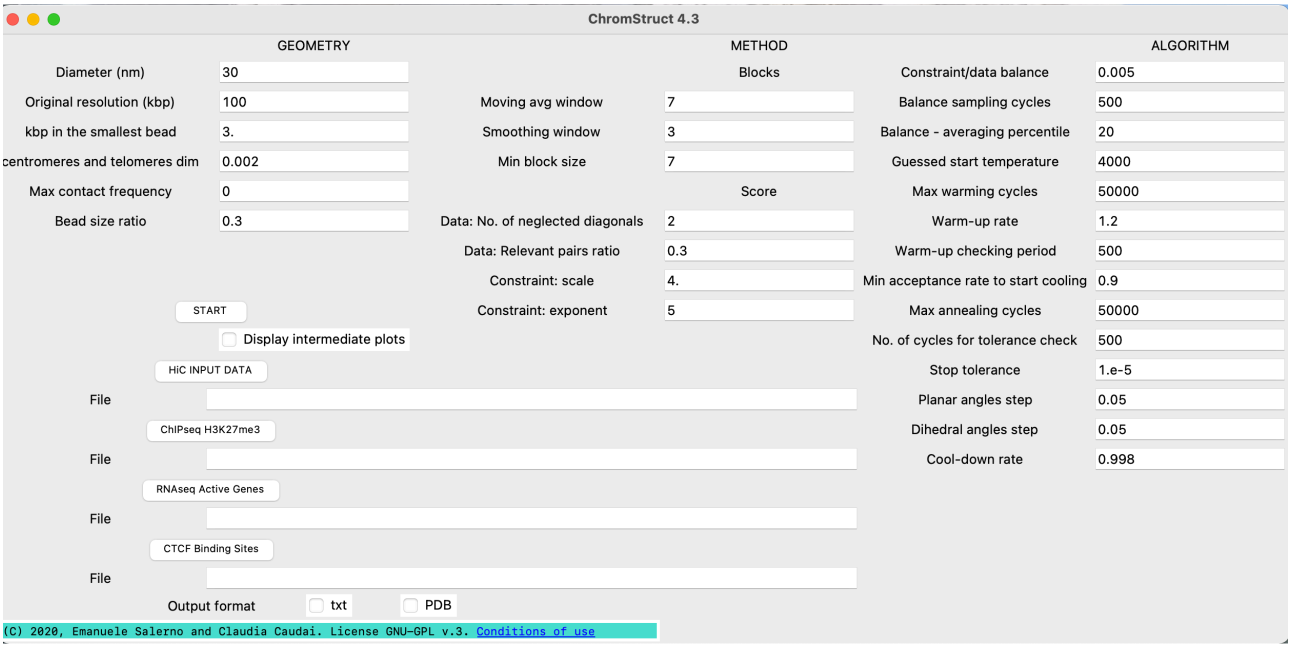

Three groups of quantities are displayed: the first includes geometrical features, the second sets up the TAD extraction and the score function, and the third is only related to the simulated annealing algorithm. All the parameters available, but in normal use only a few of them need to be tuned:

- **GEOMETRY - Original resolution:** this is the genomic resolution in kb of the data to be treated.

- **GEOMETRY - Max contact frequency:** if set to zero (the default), its value is computed as the maximum of the data matrix. In some cases, for example when different segments of the same chain are being estimated separately, it could be appropriate to set it to some fixed, user-defined value.

- **GEOMETRY - Bead size ratio:** this allows the user to tune the flexibility of the output chain. The adequacy of its choice can be evaluated *a posteriori*, by considering the biological plausibility of the output.

- **METHOD - Blocks - Min block size:** this can prevent the program from working with too small submatrices. Use cautiously.

<u>Changing the other parameters can influence the performance of the algorithm in a complicated way. We recommend to be careful when acting on them. To fully understand their significance, please refer to the commented source code and to the table at the end of this text.</u>

After setting the parameters, type the data files names in the File fields, or press the INPUT DATA button to choose the data files from the file system; their complete path then appears in the File field.

<u>*IMPORTANT:* Remember to set the variable `Original resolution` as the resolution in kb of the input contact matrix. (In the example `Original resolution=5`).</u>

Then, to start the algorithm, press the START button. If Display intermediate plots is checked, for each block, the program displays the plots of the score function values during the annealing, the number of accepted versus proposed updates during the annealing, and the estimated 3D structure of the subchain mapped onto the current data block. To continue execution, the user must first close all the graphical windows.

The code produces in output the same files already described for `ChromStruct_4.3.py` (see description above).

The two Output format checkboxes, `txt` and `PDB`, offer the possibility of choosing the format of the final estimated chain: if none or `txt` is selected, the only file `filename_<timestamp>_LastConf.txt` is stored. If `PDB` is selected, the file `filename_<timestamp>_LastConf.pdb` is stored, which can then be visualized by the standard software packages accepting the PDB format.

As denoted in GUI	Related variable	Description
**GEOMETRY**		
Diameter (nm)	`DIA`	Assumed diameter of the chromatin fiber
Original resolution (kbp)	`RIS`	Genomic size of a single locus in the original data matrix
kbp in the smallest bead	`NB`	Genomic length of a DNA chain with physical length DIA
centromeres and telomeres dimensions	`crate`	Compactness rate for centromeres and telomeres.
Max contact frequency	`NMAX`	Maximum entry in the data matrix. The default, zero, lets che code compute the value from the input data; in particular cases, for example when a chain is estimated in separate segments, the user may want to set a unique value. This is used to assign approximate sizes to the smallest-scale beads
Bead size ratio	`extrate`	Fraction of the largest principal component of a subchain centroids coordinates used to assign an approximate size to the equivalent coarser-scale bead
**METHOD**		
Moving avg window	`span`	Size of the triangular submatrix used to compute the moving average of the contact frequencies off the main diagonal of the data matrix
Smoothing window	`window`	Size of the window used to smooth the moving average function
Min block size	`minsize`	Minimum size accepted for any diagonal block extracted from the data matrix
Number of neglected diagonals	`diagneg`	Number of subdiagonals (including the main diagonal) in the data matrix to be excluded from the population of the in- contact pairs set
Relavant pairs ratio	`datafact`	Contact frequency percentile to be exceeded by any bead pair to be included in the in-contact pairs set
Constraint: scale	`scale`	Scale factor *c* in the constraint part of the score function (see reference [3])
Constraint: exponent	`exponent`	Exponent *b* in the constraint part of the score function. It must be an odd integer (see reference [3])

As denoted in GUI	Related variable	Description
**ALGORITHM**		
Constraint/data balance	`regulenergy`	A factor to balance equally the influence of data and prior knowledge in all the blocks and all scales. It sets the appropriate value for λ in all the annealing cycles. It is easily determined by trial and error. If it is not very different from its defaul value, it is not particularly critical (see reference [3])
Balance sampling cycles	`avgenergy`	Number of random configurations used to determine λ statistically. Normally, it does not need to be tuned.
Balance -averaging percentile	`percentenergy`	Score percentile used to select the random samples used to compute λ
Guessed start temperature	`Tmax`	Initial temperature set to start the annealing cycles. A too small or too large value have just the effect of slowing down the estimation
Max warming cycles	`itwarm`	Maximum number of cycles performed to evaluate the appropriate start temperature. The actual number needed is always much smaller than the default value. If the default itwarm is reached, consider looking for something wrong with the data or the parameters
Warm-up rate	`incrtemp`	Parameter used to increase the temperature until the actual annealing can start. Normally, it does not need to be tuned
Warm-up checking period	`checkwarm`	Number of periods used to check whether the right start temperature for annealing has been reached. This does not need to be tuned.
Min acceptance rate to start cooling	`muwarm`	Minimum ratio between the accepted and proposed transitions to be reached to start annealing. The default 0.9 does not need to be altered.
Max annealing cycles	`Itmax`	Maximum allowed number of annealing cycles. The actual number needed to satisfy the stop criterion is always much smaller than the default value.
Number of cycles for tolerance check	`itstop`	Cardinality of the consecutive accepted solutions set with final scores within the stop tolerance (see reference [3]). If not very different from the default, this is not a critical parameter.
Stop tolerance	`stoptol`	Stop tolerance (see row above)
Planar angles step	`RANDPLA`	Maximum random increment to be assigned to the planar angle between any two adjacent beads
Dihedral angles step	`RANDDIE`	Maximum random increment to be assigned to the dihedral angle between any two adjacent beads
Cool-down rate	`decrtemp`	Parameter used to decrease the temperature during the annealing cycles: T(n)=decrtemp*T(n-1). It is not safe to make this parameter much smaller than its default.

The picture below is a screenshot taken during a computation of `ChromStruct_4.3_GUI.py`.Top: the

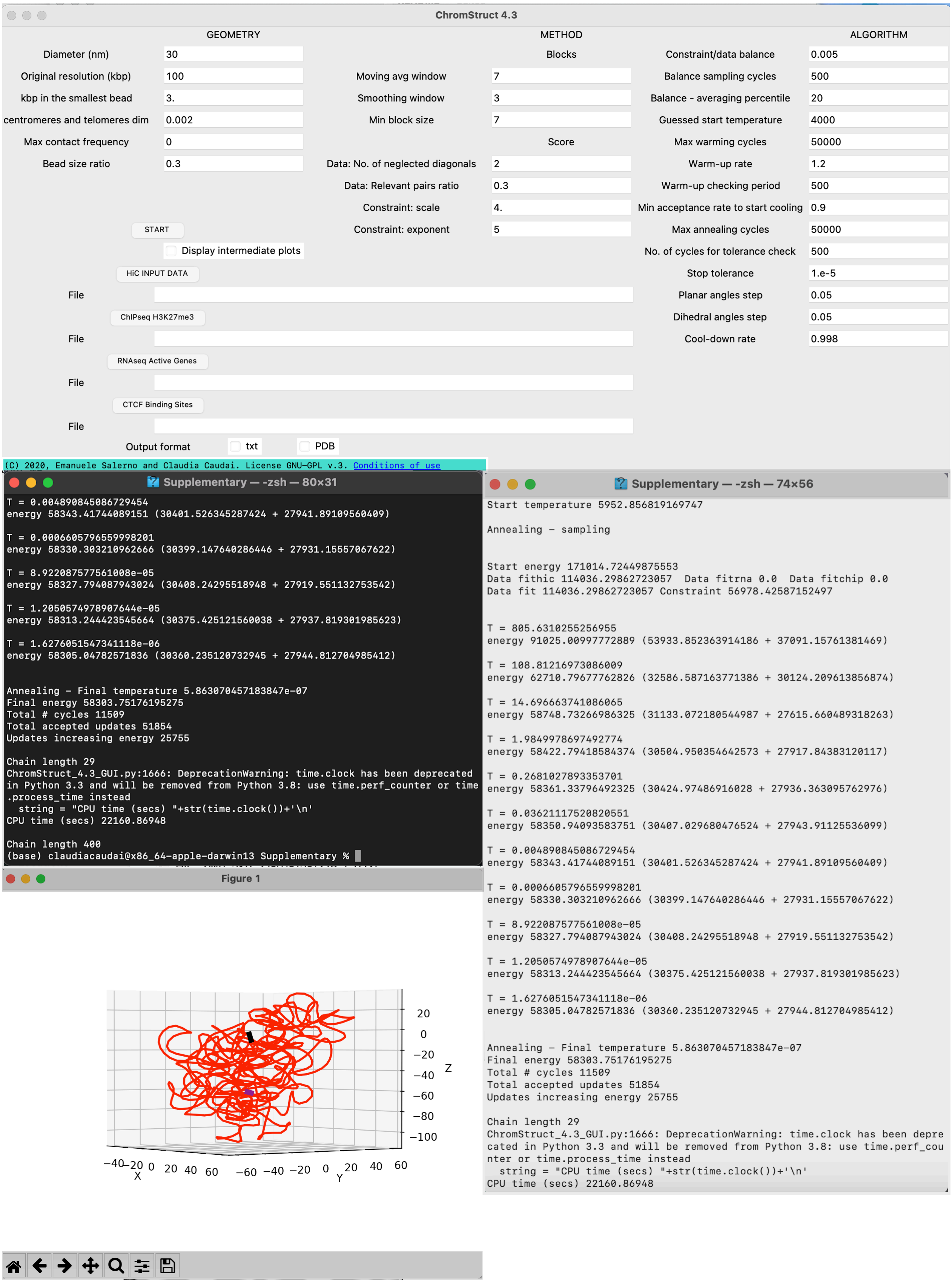

CHROMSTRUCT GUI; Bottom - right: the Python console window; left: the 3D plot of the reconstructed structure, and the directory list with the data file and the output files.

References

[1] C. Caudai, E. Salerno, M. Zoppè, A. Tonazzini, "Inferring 3D chromatin structure using a multiscale approach based on quaternions", BMC Bioinformatics, Vol. 16, 234, 2015, DOI: 10.1186/s12859-015-0667-0.

[2] Caudai, C.; Salerno, E.; Zoppè, M.; Tonazzini, A. A statistical approach to infer 3D chromatin structure. In Mathematical Models in Biology, Springer International Publishing ed.; Zazzu, V: Switzerland, 2015; pp. 325 161–171.

[3] Caudai, C.; Salerno, E.; Zoppe, M.; Merelli, I.; Tonazzini, A. ChromStruct 4: A Python Code to Estimate the Chromatin Structure from Hi-C Data. IEEE/ACM Transactions on Computational Biology and Bioinformatics 2018, pp. 1–1. doi:10.1109/TCBB.2018.2838669.

[4] Caudai, C.; Salerno, E.; Zoppe, M.; Tonazzini, A. Estimation of the Spatial Chromatin Structure Based on a Multiresolution Bead-Chain Model. IEEE/ACM Transactions on Computational Biology and Bioinformatics 2019, 16, 550–559. doi:10.1109/TCBB.2018.2791439.

[5] Mifsud, B., Tavares-Cadete, F., Young, A. *et al.* Mapping long-range promoter contacts in human cells with high-resolution capture Hi-C. *Nat Genet* 47**,** 598–606 (2015). https://doi.org/10.1038/ng.3286.

[6] Li, G.; Fullwood, M.; Xu, H.; Mulawadi, F.; Velkov, S.; Vega, V.; Ariyaratne, P.; Mohamed, Y.B.; Ooi, H.S.; Tennakoon, C.; Wei, C.L.; Ruan, Y.; Sung, W. ChIA-PET tool for comprehensive chromatin interaction analysis with paired-end tag sequencing. *Genome Biology* 2009, *11*, R22 – R22.

Previous versions of ChromStruct

CHROMSTRUCT v3.1 - Reconstruction of 3D chromatin structure from chromosome conformation capture data. E. Salerno, C. Caudai.
Software, Release 3.1, cnr.isti/2016-SW-031, 2016, DOI: 10.13140/RG.2.2.35785.13923.

CHROMSTRUCT v4.2 - Reconstruction of 3D chromatin structure from chromosome conformation capture data. E. Salerno, C. Caudai.
Software, 2018, CNR-ISTI, Pisa, 2018-388694 DOI: 10.13140/RG.2.2.26123.39208.

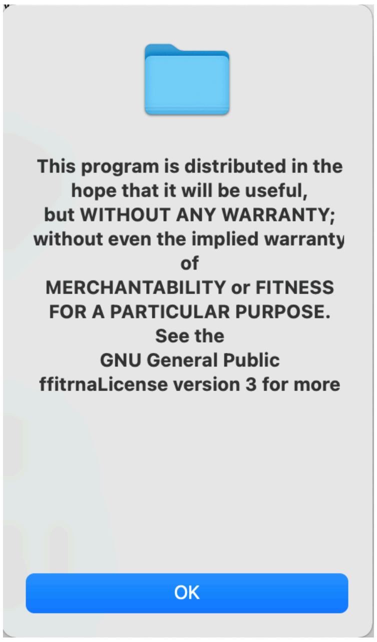

Supplement: Supplementary file 1 [file biology-10-00338-s001.zip › Supplementary/README.pdf]
